# An Overview of Self-Administered Health Literacy Instruments

**DOI:** 10.1371/journal.pone.0109110

**Published:** 2014-12-05

**Authors:** Braden O′Neill, Daniela Gonçalves, Ignacio Ricci-Cabello, Sue Ziebland, Jose Valderas

**Affiliations:** 1 Nuffield Department of Primary Care Health Sciences, University of Oxford, Oxford, United Kingdom; 2 Medical School, University of Exeter, Exeter, United Kingdom; University Of São Paulo, Brazil

## Abstract

With the increasing recognition of health literacy as a worldwide research priority, the development and refinement of indices to measure the construct is an important area of inquiry. Furthermore, the proliferation of online resources and research means that there is a growing need for self-administered instruments. We undertook a systematic overview to identify all published self-administered health literacy assessment indices to report their content and considerations associated with their administration. A primary aim of this study was to assist those seeking to employ a self-reported health literacy index to select one that has been developed and validated for an appropriate context, as well as with desired administration characteristics. Systematic searches were carried out in four electronic databases, and studies were included if they reported the development and/or validation of a novel health literacy assessment measure. Data were systematically extracted on key characteristics of the instruments: breadth of construct (“generic” vs. “content- or context- specific” health literacy), whether it was an original instrument or a derivative, country of origin, administration characteristics, age of target population (adult vs. pediatric), and evidence for validity. 35 articles met the inclusion criteria. There were 27 original instruments (27/35; 77.1%) and 8 derivative instruments (8/35; 22.9%). 22 indices measured “general” health literacy (22/35; 62.9%) while the remainder measured condition- or context- specific health literacy (13/35; 37.1%). Most health literacy measures were developed in the United States (22/35; 62.9%), and about half had adequate face, content, and construct validity (16/35; 45.7%). Given the number of measures available for many specific conditions and contexts, and that several have acceptable validity, our findings suggest that the research agenda should shift towards the investigation and elaboration of health literacy as a construct itself, in order for research in health literacy measurement to progress.

## Introduction

Health literacy- one commonly-cited definition of which is “the degree to which individuals have the capacity to obtain, process, and understand basic health information and services needed to make appropriate health decisions” [1] - is increasingly identified as an important research and policy priority [2], [3]. Using available assessment indices, a high prevalence of lower health literacy has been reported in many countries in both population-representative [4] and patient samples [5], [6]. The largest available survey from the United States, for example, was conducted by the Department of Health and Human Services in 2003, and identified 35% of the population as having ‘basic’ or ‘below basic’ levels of health literacy [7]. In an Australian sample, Barber et al [4] found a varying prevalence of ‘low’ health literacy based on their use of three separate assessment measures: between 6.8% (measured by the Test of Functional Health Literacy in Adults) [8] and 26.0% (measured by the Newest Vital Sign) [9].

Although there are many currently available indices, it has been noted that they are not all of equal quality [10]. Repeated criticisms of existing health literacy indices have noted their lack of comprehensiveness, unsuitability to specific patient populations, and psychometric weakness and heterogeneity [11], [12], [13]. The response to these criticisms, particularly in the past few years, has been to develop more indices, which have parsed health literacy into context-specific constructs [14], [15] as well as attempting more comprehensively to measure the concept in its entirety [16]. The most extensive review of available indices noted that all had significant deficiencies limiting their acceptability and generalizability [11]. While that review provided an assessment of available indices up to 2008, there have been many other indices subsequently published. In addition, the aforementioned review focused primarily on the psychometric aspects of individual indices, rather than on characteristics associated with their administration. Subsequent reviews on indices appropriate for eHealth applications [17] and diabetes [18] have been limited in scope and are useful only to select indices for these specific applications.

A key focus in recent research on health literacy measurement has been the development of self-administered indices. These do not require a trained research assistant or clinician to administer, and have been referred to in the literature as ‘indirect’ [18] or ‘self-reported’ instruments [11]. They have the advantage of potentially decreasing the burden placed on clinical practitioners while assessing health literacy, thereby reducing the resources required for research in this area. Yet, there is no available review which reports administration characteristics and critically appraises available self-administered measures, in order to support those looking to choose a measure, as well as to identify what research gaps exist in this area.

Therefore, to identify all available self-administered health literacy measurement indices, we undertook an overview of these measures. A primary aim of this study was to assist those seeking to use a health literacy index to select one that has been developed and validated for an appropriate context, as well as with desired administration characteristics. Furthermore, we aimed to inform and understand to what extent the development of additional health literacy measures is appropriate. Our purpose was to identify all available self-reported health literacy assessment measures and report their validity, their administration characteristics, and their intended uses.

## Methods

### Search strategy

We conducted systematic searches of the following electronic databases: OVID Medline (1946- April 2014), OVID Embase (1980- April 2014, OVID PsycINFO (1987- April 2014), and CINAHL (1981- April 2014). The searches were undertaken in two phases: first, on 11 September 2012, then an update search was carried out on 8 April 2014. All references were imported into EndNote X4 (Thomson Reuters, 2011).

**Figure 1:**
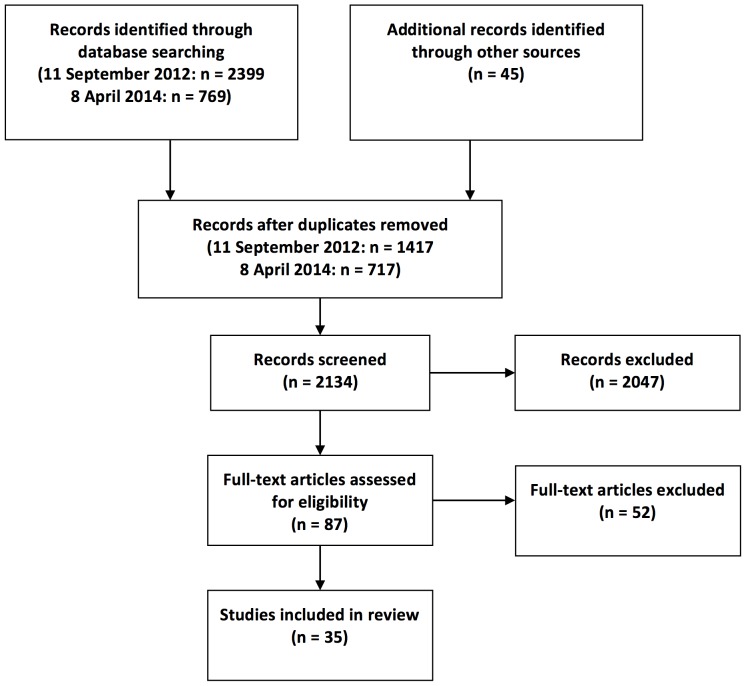
PRISMA Flow Diagram.

The search strategy was developed with a specialist librarian, with an emphasis on sensitivity to identifying studies reporting the development of condition- and specialty-specific health literacy measures, in addition to measures of general health literacy. Search terms used included literacy, health, assessment, indices, and measurement. The full search strategy is available as supporting information; see Appendix S1. Our systematic searches were augmented by ‘snowballing’ [19] from the reference lists of key identified papers, which identified 45 additional studies for title and abstract review. The PRISMA flow diagram is included as Figure 1 and the PRISMA checklist is available as supporting information (Checklist S1).

### Study selection

In order to capture the literature as broadly as possible, we included studies that reported the development and/or validation of a novel measure of health-related literacy. For inclusion, studies must have reported assessing the validity of a measure, as well as providing some description of its administration characteristics. Generic measures, as well as those focussing on more specialized health literacy such as “nutrition literacy” [20] and “eHealth literacy” [21] were included. We included only those references published in English, which reported the development and/or validation of a measure that was available in English (measures were also included if they were available in English as well as another language). Only one key reference was included for each measure (usually the first to report on a particular measure). We excluded studies that reported the development or validation of literacy measures that were not specifically related to health, as well as those that reported solely on “numeracy”, since the relationship between numeracy and health outcomes in the absence of literacy assessment remains unclear [22].

### Data extraction and synthesis

One author (BO) conducted the search; three authors (BO, DC, IRC) scanned abstracts, and selected full text articles for review. The remaining two authors (JV, SZ) contributed to developing the data extraction forms and to analysis of the extracted data. We designed and used structured forms to extract pertinent information from each article. One author extracted data from each included reference into a data extraction form in Microsoft Excel; another author (either DG or IRC) independently extracted data from each reference as well. Disagreements were resolved by consensus.

Calculations of means and standard deviations were undertaken in Microsoft Excel. Data were systematically extracted on key characteristics of the instruments: breadth of construct (‘general’ vs. ‘condition- or context- specific’ health literacy), whether it was an original instrument or a derivative, country, time for completion, number of items, age of target population (adult vs. pediatric), as well as evidence for validity. We extracted data relevant to assessing the face validity (the extent to which an index appears to measure what it is supposed to), content validity (how far items of which the index is comprised reflect the concept to be measured), and construct validity (the extent to which the index as a whole measures what it is supposed to) of included measures [22].

## Results

### Literature search

Our initial database search, carried out on 12 September 2012, identified 2399 references. An update search was carried out on 8 April 2014 identifying 769 references which had been published after the original search was carried out. Hand searching of reference lists of included papers resulted in an additional 45 references. After removing duplicate references, 2431 abstracts were scanned for inclusion and 2047 were excluded. 87 full-text articles were obtained for review, of which 52 were excluded because upon full-text review they did not report the development of a novel or newly validated self-report health literacy measure. Thus, 35 articles reporting the development and/or validation of a health literacy instrument were included (Figure 1), all of which were published during or after 2004.

### Study characteristics

#### Type of measure

Of 35 instruments included in the review, there were 27 original instruments (27/35; 77.1%) and 8 derivative instruments (8/35; 22.9%), which were either modifications or short-form versions of original instruments (Appendix S2). We classified the measures into two groups: general (stated as measuring “health literacy”, or literacy and its implications for general use of health information); and condition- or specialty-specific (stated as measuring “literacy” with a health-related prefix, such as “oral health literacy” [23] or “colon cancer literacy” [26]). Most measures were classified as general (22/35; 62.9%%), while the remainder (13/35; 37.1%) were classified as condition - or specialty specific. Indices were available for a variety of conditions, such as Human Immunodeficiency Virus (HIV) infection [24], cancer [25], [26], as well as specific indications such as eHealth [21] (Table 1).

**Table 1:** Indications for general and content- or context- specific measures.

Indication	Measures
General health literacy	3 questions [27]; NAAL HL [28]; SILS [29]; CCHL [30]; CHC [31]; HLSI [16]; METER [32]; Talking touchscreen [33]; Graph literacy [34]; Health LiTT [35]; CHL [36]; TAIMI [37]; MHLS [38]; Canadian high school student measure [39]; HLSI short form [40]; SDPI-HH HL [41]; Massey 2012 measure [42]; CAHPS Item Set [43]; AAHLS [44]; HeLMS [45]; HLQ [46]
Dental/oral health literacy	HeLD [23]; Harper 2014 measure [47]
Diabetes literacy	FCCHL [48]
Cancer literacy	SIRACT [26]; CMLT-L/CMLT-R [25]
Mental health literacy	Reavley 2014 measure [49]
Nutrition literacy	FLANKK [50]; NLAI [20]
Hospital literacy	HCAHPS Item Set [51]
HIV literacy	HIV-HL [24]
Medication literacy	MedLitRxSE [52]
Colon cancer literacy	ACCL [14];
Intellectual disability literacy	ILDS [53];
eHealth literacy	eHEALS [21];

#### Country of origin

Most of the available instruments to measure health literacy have been validated in the United States (22/35; 59.5%), with Australia and Japan being the next most frequent country of origin of health literacy indices (both contributed 4/35; 11.4%). Two indices were validated in each of the following countries: the United Kingdom, Canada, and Germany. There were two measures initially validated in multiple countries: the ‘Graph literacy’ scale (in Germany and the United States) [34] and the Intellectual Disability Literacy Scale (ILDS; validated in the United Kingdom, India, China, and Singapore) [53] (Table 2).

**Table 2:** Country of origin of measures.

Country of origin	Measures
United States	3 questions [27]; NAAL HL [28]; SILS [29]; HLSI [16]; METER [32]; Talking touchscreen [33]; Health LiTT [35]; HLSI short form [40]; SDPI-HH HL [41]; Massey 2012 measure [42]; CAHPS Item Set [43]; SIRACT [26]; CMLT-L/CMLT-R [25]; FLANKK [50]; NLAI [20]; ACCL [14]; MedLitRxSE [52]; HCAHPS Item Set [51]
Australia	HeLMS [45]; HLQ [46]; HeLD [23]; Reavley 2014 measure [49]
Japan	FCCHL [48]; CCHL [30]; CHL [36]; TAIMI [37]
Canada	eHEALS [21]; Canadian high school student measure [39]
United Kingdom	ILDS [53]; AAHLS [44]
China	ILDS [53]
Germany	CHC [31]
India	ILDS [53]
Korea	MHLS [38]
Singapore	ILDS [53]

#### Setting

Most included studies reported the setting from which participants were recruited for validation. About a third of the instruments were validated in populations recruited from secondary and specialty care settings (12/35; 34.2%), while a quarter of studies recruited in primary care settings (8/35; 22.9%). The remainder of validation studies recruited from diverse non-clinical settings (17/35; 48.6%), such as schools and community centres, and shopping malls. Two studies [39], [47] reported validating general health literacy measures through online surveys and did not provide information about the characteristics of the subjects recruited. Three studies [41], [46], [52] recruited participants from more than one setting (Appendix S2).

#### Age of participants

The majority of available indices were initially studied and validated with populations between 18 and 65 years old. The mean age of participants in included studies ranged from 18 to 76 years. There were six indices validated in <18 year old populations: the eHealth Literacy Scale (eHEALS) [21], the Food Label Literacy for Applied Nutrition Knowledge scale (FLLANK) [50], the All Aspects of Health Literacy Scale (AAHLS) [44], and three unnamed measures: one for adolescents [42], one for Canadian high school students [31], and one to assess mental health literacy [49]. One of these, the eHEALS, is suggested by the authors to be valid for all ages.

#### Psychometric characteristics

16 indices were assessed in included studies as having adequate face, content, and construct validity (16/35; 45.7%) (Table 3). 18 indices had adequate face and content validity, but construct validity was not assessed in the study included in this review (18/35; 51.4%). One index (validated in one of the largest studies included in this review, which included 6083 participants) (1/35; 2.9%) established face validity only [37]. There were no clear differences between the validity of general indices versus content- and context- specific (Table 3). No included studies reported that sensitivity to change in health literacy level over time could be measured using the instrument, but test-retest reliability was reported as being adequate in four measures [21], [45], [50], [53]. The authors of one measure noted that although test-retest reliability had not been addressed in their study, that they intended to assess this in a future study [46].

**Table 3:** Psychometrics of general and context- or content- specific indices.

Levels of validity addressed	Number (%) of general measures at each level	Number (%) of context- or content- specific indices at each level
Face only	1 (4.5)	0 (0)
Face and content	11 (50.0)	6 (46.2)
Face, content, and construct	10 (45.5)	7 (53.8)

#### Time to administer/number of items

Only 16 studies (16/35; 45.7%) reported how long it takes to administer the instrument being reported; this ranged from 2 minutes (Medical Term Recognition Test; METER) [32] to 70 minutes (Cancer Message Literacy Test- Listening/Cancer Literacy Message Test- Reading; CMLT-L/CMLT-R) [25]. Overall, health literacy indices required on average 19.1 (SD 19.6) minutes to administer, although there are 7 available measures that can be administered in 5 minutes or less [9], [21], [27], [29], [32], [36], [40] (Appendix S2). General measures took an average of 22.3 minutes (SD 26.1) to complete, while condition- and specialty- specific measures took an average of 20.8 minutes (SD 22.5). 32/35 (91.4%) indices reported the number of items included, which varied from 1 [29] to 80 [32] (SD 20.5) (Appendix S2).

## Discussion

### Statement of principal findings

This review reports the administration characteristics and validity of 35 health literacy measures, and aims to assist selection of an appropriate index. The majority of included indices have been developed and validated in the United States. There is a clear trend towards the development of more measures in recent years. The instruments identified in this review are mostly intended for adults (with only six available for pediatric populations) and have been primarily tested in non-clinical settings. Several instruments take less than 5 minutes to complete, and there are many with adequate validity.

Low health literacy is strongly associated with worse health outcomes [54]. In order to improve the provision of healthcare for patients with low health literacy, it has been suggested that we need to have appropriate measurement tools to identify these individuals at clinical and population levels. Several authors have proposed that new health literacy assessment measures are required [11], [12], [55], [56] yet we have identified 35 indices, validated for use in a wide variety of populations. While it is understandable that no single assessment measure has been able to represent a complex construct such as health literacy in its entirety, there are additional aspects to the construct itself that are inherent to the way in which it is assessed. Our review is the first to use systematic overview methodology to review the administration, development, and grouping of generic and condition- or context- specific self-report measures. We used a sensitive search strategy to report the breadth of health-related literacy assessment, building upon an earlier review of the psychometric properties of 19 health literacy instruments [11].

### Conceptual implications for health literacy

The measures included in this review represent a cross-sectional evaluation of 10 years of health literacy measurement research. We focused on self-report measures because these require fewer resources than those for which examiners (or clinicians) must be trained and provided. Consequently, we believe there will be more of a future in self-administered measures, as they are more scalable and can more easily be incorporated into online surveys, which are becoming more prevalent in health research [57], [58], [59].

It has been noted that there is underlying conceptual disagreement about what health literacy is and that this may be contributing to an “unstable foundation” in research in this area [60]. Our review identified two broad classes of measures, each of which represented about half of the available self-report measures. While ‘general’ measures seek to measure the construct in its entirety, ‘condition- or context- specific’ measures are focused on a specific area in which health literacy is used by individuals in the course of making health decisions. These two streams of research diverge to some extent in how they conceptualize health literacy; while the development of ‘general’ measures seeks to better measure (and thereby more comprehensively define) the entire construct based on some *a priori* definition of health literacy, ‘condition- or context- specific’ indices are developed in the course of identifying a particular area in which the authors (presumably) believe it will be more beneficial to measure *specific* deficits that can be ameliorated. Existing research has not found clear patterns of effectiveness in interventions that are designed to mitigate the detrimental effects of low health literacy [60], [61], [62].

Complimentary to ‘mitigating’ the negative effects is the idea of ‘improving’ health literacy, which is widespread throughout available policy documents [63], [64], [65], [66]. Yet, there is little evidence on how to do this. The separate streams of research identified in this overview may offer some promise for developing a way of going about improving people's health literacy; either ‘general’ health literacy should be targeted, or some context-specific operationalization of health literacy should be addressed. None of these measures demonstrated sensitivity to change, so even if it was possible to ‘improve’ health literacy, it appears no current measure would be appropriate to assess this.

### Limitations

This study has identified that new measures are being developed and published frequently. One important limitation is therefore that there are other measures which have been developed and published since the search was carried out which have not been included. In addition, given the clinical heterogeneity and diversity of health literacy measurement, it is possible that relevant studies available at the time the search was carried out have not been retrieved. In particular, since only one reference was included for each measure, it is probable that many of these measures were further validated in subsequent studies. Although the risk of bias was not determined for each individual study, the quality of *instruments* reported in included studies was assessed; since the primary aim of this study was to assess the quality of these measures, it was felt that being inclusive regarding studies would allow this study to more adequately represent the available literature. Given that one of the conclusions of this study is that the need for additional health literacy measures should be questioned, this selection bias serves only to further the point that there are already many high-quality available general and specific indices available. Limiting this study to self-administered measures has the advantage of providing those looking for one of these measures an accessible, clear reference to guide selection of an index of this type, however it excludes many other health literacy measures, which may be of lesser quality. Conclusions of a review encompassing all the measures may prove less optimistic about the quality of available indices for health literacy measurement, which is what the most comprehensive available review including these measures identified [11].

### Relevance to an ongoing research agenda for health literacy measurement

Researchers and clinicians alike need to consider practicalities of administration, including how long a measure takes to complete, whether it is suitable for self-completion (either online or with paper and pencil), and in what other circumstances and populations it has been used. Our review draws attention to the fact that several health literacy assessments require a great deal of time, potentially exceeding the average length of a primary care consultation. In a large European study, for example, Deveugele et al [67] found that the average length of a primary care consultation was 10.7 (6.7) minutes. In the present study, the average time required to administer a clinical or clinical/research health literacy assessment in this review was 20.7 (23.6) minutes. Since primary care was a common location in which these indices were developed and validated, it is critical that future research in this area is attentive to the reality of busy clinical practice. Our review demonstrates that currently, health literacy assessment using most available tools is impractical in clinical practice due to the time required, despite authors' suggestions that some of these measures should be incorporated into consultations. Despite the association between low health literacy and poorer outcomes, there is no evidence that health literacy screening has an effect on health outcomes [68]. It seems unlikely that health literacy assessment will become a fixture of clinical practice. Resources might be better allocated to developing interventions to mitigate the effect of low health literacy on health outcomes, for which there is already a strong evidence-base.

Almost three quarters of available health literacy indices included in this review have been developed and validated in populations in the United States. Indices from the United States may not be fully transferable to another health system, although it should be noted that this review only included English language papers. Again, in contrast to proposed research agendas for health literacy [55], [56] it is unclear whether this is best dealt with by undertaking validation of existing indices in other countries and health systems, as opposed to developing new indices to address these issues.

Many successful interventions intended to mitigate the effects of low health literacy on outcomes have resulted in mixed effects [60], [61], [62]. Some have improved health outcomes, such as end-of-life care preferences [69] whereas others have had no effect on other outcomes such as hemoglobin A1c in patients with type II diabetes [70]. Although it is somewhat unclear what benefit the development of additional health literacy assessment measures could provide, one key deficiency is that in order to move forward in ‘improving’ people's health literacy, either new indices must either be developed or existing indices tested to determine if they are sensitive to change over time. It will not be possible to ascertain if it is even possible to ‘improve’ people's health literacy until there are measures which have been shown to be sensitive to change in this regard.

## Conclusion

Asking the right questions is critical to effective research, and doing so is necessary to eliminate “research waste” [71]. There are currently 35 self-report health literacy measures available, validated in a variety of contexts and intended for diverse applications. Since many already have adequate validity, it should be identified whether existing measures are sensitive to change as a result of improved health literacy. It is probable that some of these existing measures may be adequate for this purpose, but if not, this is a key deficiency that would suggest the development of new measures Further conceptual work on health literacy is necessary to understand whether it is a static or dynamic construct. These findings will influence the research agenda for whether it is necessary to develop new measures, or to expand the use of existing ones.

## Supporting Information

Checklist S1 **PRISMA Checklist.**
(DOC)

Appendix S1 **Search strategy for MEDLINE.**
(DOCX)

Appendix S2 **Characteristics of included indices.**
(DOCX)
