# Supplementary material for: An Overview of Self-Administered Health Literacy Instruments
Source: PLoS One. 2014 Dec 5;9(12):e109110. doi: 10.1371/journal.pone.0109110 (PMC4257499; doi:10.1371/journal.pone.0109110)
Supplement: Appendix S1 — Search strategy for MEDLINE. (DOCX) [file pone.0109110.s002.docx]

**Appendix S1:** Sample search strategy (MEDLINE)

Searches carried out on 12 September 2012 and 8 April 2014

1: health literacy.mp.

2: assessment.mp.

3: measure*.mp.

4: tool.mp.

5: indices.mp.

6: instrument.mp.

7: 2 or 3 or 4 or 5 or 6

8: 1 and 7
