# Supplementary material for: An Overview of Self-Administered Health Literacy Instruments
Source: PLoS One. 2014 Dec 5;9(12):e109110. doi: 10.1371/journal.pone.0109110 (PMC4257499; doi:10.1371/journal.pone.0109110)
Supplement: Appendix S2 — Characteristics of included indices. (DOCX) [file pone.0109110.s003.docx]

Appendix S2: Health literacy indices identified in the included studies (in ascending order by year of publication)

| **First author, year** | **Name of measure (abbreviation)** | **Original or derivative measure?** | **Country** | **Sample size** | **Setting in which it was validated (Primary care; Secondary care/other clinical practice; Non-clinical)** | **Time required to administerminutes (standard deviation)** | **Number of items included in measure** | **Validity**  **(Face, Content, Construct)** | **Test – retest**  **reliability** | **What type of measure is it (General; Condition- or context- specific)** |
| --- | --- | --- | --- | --- | --- | --- | --- | --- | --- | --- |
| Chew, 2004 | 3 questions | Original | United States | 332 | Secondary care/other clinical practice | Not reported (assumed  < 5) | 3 | Face, Content, Construct | Not reported | General |
| Kutner, 2006 | National Assessment of Adult Literacy, Health Literacy (NAAL HL) | Original | United States | 19000 | Non-clinical | Not reported | 28 | Face, Content | Not reported | General |
| Morris, 2006 | Single Item Literacy Screener (SILS) | Derivative (3 questions) | United States | 999 | Primary care | Not reported (assumed  < 5) | 1 | Face, Content, Construct | Not reported | General |
| Norman, 2006 | eHealth Literacy Scale (eHEALS) | Original | Canada | 664 | Non-clinical | 3 | 8 | Face, Content | Intraclass correlation coefficient = 0.49 | Condition- or context- specific (eHealth) |
| Agre, 2007 | Stieglitz Informal Reading Assessment of Cancer Text (SIRACT) | Original | United States | 45 | Secondary care/other clinical practice | 10 to 20 | Not reported | Face, Content | Not reported | Condition- or context- specific (cancer) |
| Ishikawa, 2008 | Functional, Communicative, Critical Health Literacy Scale  (FCCHL) | Original | Japan | 138 | Secondary care/other clinical practice | Not reported | 14 | Face, Content, Construct | Not reported | Condition- or context- specific (diabetes) |
| Ishikawa, 2008 | Communicative and Critical Health Literacy Scale (CCHL) | Derivative (FCCHL) | Japan | 229 | Non-clinical | Not reported | 5 | Face, Content, Construct | Not reported | General |
| Steckelberg, 2009 | Critical Health Competence Test (CHC) | Original | Germany | 107 | Non-clinical | 90 | 72 | Face, Content | Not reported | General |
| McCormack, 2010 | Health Literacy Skills Instrument (HLSI) | Original | United States | 889 | Non-clinical | 38 | 25 | Face, Content, Construct | Not reported | General |
| Rawson, 2010 | Medical Term Recognition Test (METER) | Original | United States | 155 | Secondary care/other clinical practice | 2 | 80 | Face, Content, Construct | Not reported | General |
| Yost, 2010 | Talking touchscreen | Original | United States | 610 | Primary care | 18 | 30 | Face, Content, Construct | Not reported | General |
| Galesic, 2011 | Graph literacy | Original | Germany,  United States | German pilot study: 120  German sample: 495  United States sample: 492 | Non-clinical | 10 | 13 | Face, Content | Not reported | General |
| Hahn, 2011 | Health Literacy Assessment Using Talking Touchscreen Technology (Health LiTT) | Derivative (Talking touchscreen) | United States | 608 | Primary care | 18 | 30 | Face, Content, Construct | Not reported | General |
| Ishikawa, 2011 | Communicative Health Literacy (CHL) | Derivative (FCCHL) | Japan | 143 | Secondary care/other clinical practice | 5 to 10 | 5 | Face, Content | Not reported | General |
| Scior, 2011 | Intellectual Disability Literacy Scale (IDLS) | Original | United Kingdom,  India,  China,  Singapore | Pilot study: 114; At 2/3 weeks follow up: 300  Main study: 1376 | Non-clinical | Not reported | 44 | Face, Content, Construct | Kappa >.6 for four subscales; >.7 for ten subscales | Condition- or context- specific (intellectual disability) |
| Takahashi, 2011 | Test for Ability to Interpret Medical Information  (TAIMI) | Original | Japan | 6083 | Not reported; online survey | Not reported | 7 | Face | Not reported | General |
| Tsai, 2011 | Mandarin Health Literacy Scale (MHLS) | Original | Korea | 323 | Non-clinical | 25 | 50 | Face, content | Not reported | General |
| Begoray, 2012 | Canadian high school student measure | Original | Canada | 229 | Not reported | Not reported | 18 | Face, Content, Construct | Not reported | General |
| Bann, 2012 | Health Literacy Skills Instrument (HLSI): Short form version | Derivative  (HLSI) | United States | 889 | Non-clinical | 5 to 10 | 10 | Face, content, construct | Not reported | General |
| Brega, 2012 | Special Diabetes Program for Indians Healthy Heart Health Literacy (SDPI-HH HL) | Derivative (3 questions; General numeracy test; TOFHLA)^[[1]](#footnote-1)^ | United States | 3033 | Primary care; secondary care/other clinical practice | Not reported | Not reported | Face, content, construct | Not reported | General |
| Massey, 2012 | No name | Derivative (YAHCS; eHEALS; HINTS)^[[2]](#footnote-2)^ | United States | 1208 | Non-clinical | Not reported | Not reported | Face, content | Not reported | General |
| Mazor, 2012 | Cancer Message Literacy Test-Listening; Cancer Message Literacy Test-Reading (CMLT-L/CMLT-R) | Original | United States | Pre-pilot study: 7 Pilot study: 79 | Secondary care/other clinical practice | 70 | CMLT-L: 48  CMLT-R: 21 | Face, Content | Not reported | Condition- or context- specific (cancer) |
| Pendlimari, 2012 | Assessment of Colon Cancer Literacy (ACCL) | Original | United States | 61 | Secondary care/other clinical practice | Not reported | 10 | Face, content, construct | Not reported | Condition- or context- specific (colon cancer) |
| Reynolds, 2012 | Food Label Literacy for Applied Nutrition Knowledge (FLANKK) | Original | United States | 499 | Non-clinical (school) | 15 | 10 | Face, content | Intra-class correlation coefficient 0.61-0.73 | Condition- or context- specific (nutrition) |
| Sauceda, 2012 | Medication Literacy Assessment in Spanish and English (MedLitRxSE) | Original | United States | 181 | Primary care; non-clinical | 22 | 14 | Face, content, construct | Not reported | Condition- or context- specific (medication literacy) |
| Weidmer, 2012a | HCAHPS Item Set for Addressing Health Literacy | Original (to be co-administered with another measure: HCAHPS^[[3]](#footnote-3)^) | United States | 1013 | Secondary care/other clinical practice | Not reported | 62 | Face, content, construct | Not reported | Condition- or context- specific (hospital medicine) |
| Weidmer, 2012b | CAHPS Item Set for Addressing Health Literacy | Original | United States | 601 | Primary care | Not reported | 22 | Face, content | Not reported | General |
| Chinn, 2013 | All Aspects of Health Literacy Scale (AAHLS) | Original | United Kingdom | 146 | Primary care | 7 | 14 | Face, content, construct | Not reported | General |
| Gibbs, 2013 | Nutrition Literacy Assessment Instrument (NLAI) | Original | United States | 178 dietitians26 patients | Secondary care/other clinical practice (dietitian's outpatient clinics) | 8.4 (3.0) | 35 | Face, content | Not reported | Condition- or context- specific (nutrition) |
| Jordan, 2013 | Health Literacy Management Scale (HeLMS) | Original | Australia | 683 | Non-clinical | Not reported | 37 | Face, content | Intra-class correlation coefficient 0.73-0.96 | General |
| Osborne, 2013 | Health Literacy Questionnaire (HLQ) | Original | Australia | 1039 | Primary care; secondary care/other clinical practice; non-clinical | Not reported | 44 | Face, content, construct | Not reported (will be addressed in future study) | General |
| Ownby, 2013 | HIV-Related Health Literacy Scale (HIV-HL) | Original | United States | 120 | Secondary care/other clinical practice | 10 to 15 | 20 | Face, content, construct | Not reported | Condition- or context- specific (HIV) |
| Harper, 2014 | No name | Original | United States | 144 | Non-clinical (online study w/ students) | Not reported | 51 | Face, content | Not reported | General |
| Jones, 2014 | Health Literacy in Dentistry (HeLD) | Derivative (HeLMS) | Australia | 209 | Non-clinical | Not reported | 29 | Face, content, construct | Not reported | Condition- or context- specific (oral health) |
| Reavley, 2014 | No name | Original | Australia | 6019 patients, 1536 health profess-sionals | Non-clinical | Not reported | 54 | Face, content | Not reported | Condition- or context- specific (mental health) |

1. 3 questions: Chew et al 2004 measure (see above, p. 1); General numeracy test (Lipkus IM, Samsa G, Rimer BK. General Performance on a Numeracy Scale among Highly Educated Samples*.* *Medical Decision Making*. 2001;21(1):37-44); TOFHLA: Test of Functional Health Literacy in Adults (see above). [↑](#footnote-ref-1)
2. YACHS: Young Adult Health Care Survey (Bethell C, Klein J, Peck C. Assessing health system provision of adolescent preventive services: the Young Adult Health Care Survey*.* *Medical Care*. 2001;39(5):478-490.); eHEALS: eHealth Literacy Scale (see above, p. 2); HINTS: Health Information National Trends Survey (Nelson D, Kreps G, Hesse B, Croyle R, Willis G, Arora N, Rimer B, Vish Viswanath K, Weinstein N, Alden S. The health information national trends survey (HINTS): Development, design, and dissemination*.* *Journal of Health Communication*. 2004;9(5):443-460.) [↑](#footnote-ref-2)
3. HCAHPS: Consumer Assessment of Healthcare Providers and Systems Hospital Survey (Goldstein E, Farquhar M, Crofton C, Darby C, Garfinkel S. Measuring hospital care from the patients' perspective: An overview of the CAHPS® hospital survey development process*.* *Health Services Research*. 2005;40(62):1977-1995.) [↑](#footnote-ref-3)
